# The Design of Terahertz Frequency Quadruplers Based on Discrete Schottky Diodes

**DOI:** 10.3390/mi13010069

**Published:** 2021-12-31

**Authors:** Yuhang Li, Dehai Zhang, Jin Meng, Haotian Zhu, Siyu Liu

**Affiliations:** 1 Key Laboratory of Microwave Remote Sensing, National Space Science Center, Chinese Academy of Sciences, Beijing 100190, China; liyuhang17@mails.ucas.ac.cn (Y.L.); mengjin@mirslab.cn (J.M.); zhuhaotian@mirslab.cn (H.Z.); liusiyu16@mails.ucas.ac.cn (S.L.); 2 School of Electronic, Electrical and Communication Engineering, University of Chinese Academy of Sciences, Beijing 100049, China

**Keywords:** frequency quadrupler, gradient line, idle loop, schottky varactor

## Abstract

On the basis of the W-band power source, a single-stage frequency quadrupler method was used to implement two 335 GHz frequency quadruplers. The two frequency quadruplers adopted a traditional binomial matching structure and an improved gradient line matching structure, respectively. An idle loop was added to the overall circuit in the design of the DC filter and low-pass filter. The improved gradient line matching structure reduced the circuit length while increasing the bandwidth, effectively reducing the power loss on the transmission line. A micro-strip circuit was fabricated with a 50 μm thick quartz circuit and was mounted onto a split waveguide block. The results showed that the output power of the quadrupler with the improved matching structure was better than that of the quadrupler with the conventional matching structure. The peak output power of the improved frequency quadrupler was 4.75 mW at 333 GHz when driven with 200 mW. In contrast, this improved structure broadened the bandwidth by 8 GHz and reduced the length of the substrate by 0.607 mm, effectively reducing the length of the traditionally designed circuit by 11.5%.

## 1. Introduction

The terahertz band, which spans the gap between infrared and millimeter waves, has been a very valuable research field in recent years. It has broad application prospects in radio astronomy, broadband communication, global water cycle observations, object imaging, marine environmental monitoring, etc. [1,2,3,4,5]. The contemporary development of terahertz systems is mainly limited by the generation of terahertz waves. Solid-state frequency multipliers, based on planar Schottky diode technology, have the advantages of high integration, stable operation, and reasonable cost. It has become the main method to obtain terahertz signal sources [6]. In this method, the nonlinear effects of solid-state devices are used to generate each harmonic of the fundamental input wave, and the required harmonic components are extracted by filters, so as to reduce the main oscillation frequency of the system. Through the research in recent years, many results have been achieved in the field of solid-state frequency multiplication [7,8,9,10,11,12].

As one of the atmospheric transmission windows in the terahertz band, the band near 330 GHz has potential application prospects in the terahertz communication field, and it is also an important frequency point in the remote sensing field. Therefore, a 335 GHz frequency multiplier was designed and implemented in this study. The design considers the front drive, overall link compactness, cost and process. In this study, the frequency multiplier was realized by single-tube single-channel quadrupling, which avoids the risk of multi-level frequency multiplication mismatch. Through the analysis and design of the idle circuit, the short circuit of the unnecessary harmonic idle circuit was realized. The gradient line structure was applied to the diode matching circuit, which not only facilitated the assembly of the diode, but also broadened the working bandwidth, to a certain extent, and reduced the power loss. By comparing the measured results of the frequency multiplier to the traditional binomial matching structure, the superiority of the gradient line matching structure is verified.

## 2. Quadrupler Design

### 2.1. Idle Loop Theory Analysis and Design

Frequency multipliers can be divided into resistive frequency multipliers and capacitive frequency multipliers, according to the frequency multiplication mode. Resistive frequency multipliers have a lower efficiency but a wider bandwidth. The main target of this design is to obtain a higher frequency doubling efficiency and output power. The frequency multiplier was realized using the capacitive frequency multiplication method, and the varactor diode was selected as its core non-linear device. Theoretically, when designing a capacitive frequency multiplier, an idle loop is required to achieve the desired harmonic power output. In order to achieve the fourth harmonic power output, an operating circuit with a harmonic number lower than four (in addition to the fourth harmonic) needs to be set up in the circuit [13]. The function of the idle circuit in the frequency multiplier is to send the harmonic power generated by the diode back to the diode, and then convert the low-order harmonic power into high-order harmonic power through its nonlinear characteristics. In summary, its main function is to perform energy conversions. Although this increases the complexity of the circuit, it can effectively improve the efficiency and output power of frequency multipliers.

**Figure 1:**
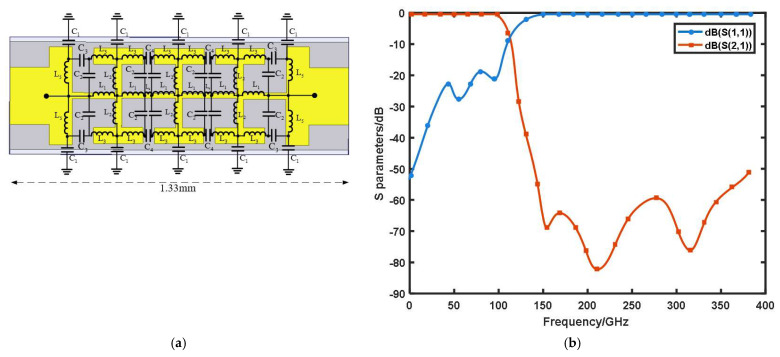
(**a**) Low–pass filter structure; (**b**) simulation results of low-pass filter.

In this design, we adopted a DC bias filter and a low-pass filter to add each loop to the entire circuit. The low-pass filter was designed with a compact resonant cell structure. By establishing the fringe capacitance from the microstrip to the metal wall, the value of the grounding capacitance, $C_{1}$, was greatly reduced. By changing the value of $L_{1}-L_{5}$ to adjust the size of the resonator, the value of $C_{2}-C_{4}$ also changed at this time. These capacitor values mainly affected the suppression degree of the filter. From the resonance frequency $f_{0}=1/{\left(\mathrm{LC}\right)}^{1/2}$, it could be observed that the adjustment of these values improved the resonance frequency to a certain extent. Accordingly, the corresponding three-dimensional electromagnetic simulation model was established, as shown in Figure 1a. The S-parameter results are shown in Figure 1b. It can be seen that the return loss was less than 0.3 dB and the insertion loss was less than −19 dB in the 75–95 GHz band, whereas the return loss was less than −50 dB and the insertion loss was less than 0.2 dB in the 206–380 GHz band. The impedance value of the second harmonic was (0.08 + 1.02i) Ω@226 GHz. The real part was close to 0, which indicated that the entire idle loop was lossless; however, the non-zero imaginary part indicated that the idle loop successfully participated in the entire frequency quadrupler circuit.

**Figure 2:**
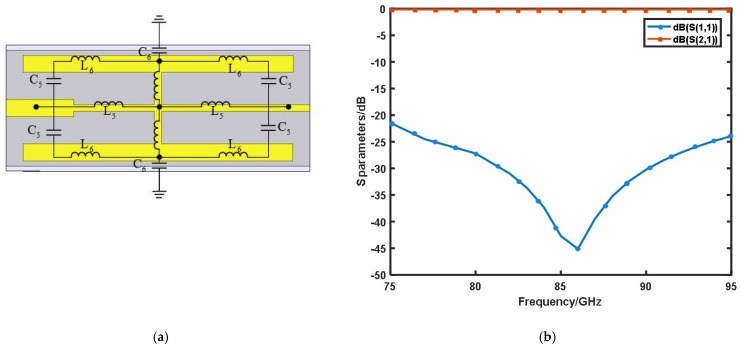
(**a**) DC filter structure; (**b**) simulation results of the DC filter (S11 > 21 dB, S21 < 0.2 dB at 75–95 GHz).

In the design of the DC bias filter, we also adopted a compact resonant unit structure, as shown in Figure 2a. The narrow metal conduction band was equivalent to the inductance, and the gap between the conduction bands was equivalent to the capacitance. The required passband and stopband could be achieved by changing the length and width of each section by adjusting the capacitance and inductance values. It can be seen in Figure 2b that the filter achieved a good blocking effect in the input frequency band. The isolation was greater than 20 dB and the return loss was less than 0.2 dB in the 75–95 GHz band.

The results of the simulation impedance and S-parameters showed that the second and third harmonic idle loops were short-circuited by using two circuit structures. This satisfied the isolation requirements of the other idle harmonics in the whole fourth frequency multiplier circuit [14].

### 2.2. Matching Circuit Improvement

In the design of frequency multipliers, it is inevitable that there will be a mismatch in the load of the microwave transmission line or the connection of the transmission lines with different characteristic impedances. The reflection caused by the above situation will increase the loss, and reduce the power capacity and the efficiency. This problem can be solved by connecting an impedance converter. In a frequency multiplier, this is generally composed of one or several transmission lines with different characteristic impedances, which is the matching circuit part. The main purpose of the matching circuit is impedance matching, to avoid unnecessary power loss.

In order to increase the total frequency band of the converter, the characteristic impedance, Zn, or reflection coefficient, ρn, of each section should be selected reasonably. Regarding the specific choice of ρn, it can be determined according to the binomial distribution or the Chebyshev distribution. The Chebyshev converter was designed by making Γ(θ) equal to the Chebyshev polynomials, because the Chebyshev polynomials have the best properties required for this type of converter. The passband response of the binomial matched converter was the flattest. In summary, the Chebyshev distribution in the text refers to an equal ripple bandpass response, where the optimum bandwidth was obtained at the cost of ripples within the passband. If the section of the stepped Chebyshev converter increases to infinity and the length of each section decreases indefinitely, the converter changes from discontinuous step transition to gradual transition [15].

The natural logarithm of the characteristic impedance change in the gradient line is as follows:

(1) $$\ln Z\left(z\right)=\frac{1}{2}\ln Z_{0}Z_{L}+\frac{\Gamma_{0}}{\cosh A}A^{2}\varphi \left(\frac{2z}{L}-1,A\right),\;\;\;0\leq z\leq L$$

In Equation (1), $Z_{0}$ is the impedance when viewed from the transmission line towards the matching network, $Z_{L}$ is the load impedance, and *L* is the number of impedance converter sections. 

The function φ(x,A) is defined as follows:

(2) $$\varphi \left(x,A\right)=-\varphi \left(-x,A\right)=\int_{0}^{x}\frac{I_{1}\left(A\sqrt{1-y^{2}}\right)}{A\sqrt{1-y^{2}}}\mathrm{dy},\;\;\;\;\left|x\right|\leq 1$$

According to the small reflection theory, the total reflection coefficient of the graded transmission line is the sum of all local reflections with appropriate phase shifts. Using the results of [16], the reflection coefficient is as follows:

(3) $$\Gamma \left(\theta \right)=\Gamma_{0}e^{-j\beta L}\frac{\cos\sqrt{{\left(\beta L\right)}^{2}-A^{2}}}{\cosh A},\;\;\;\;\;\beta L>A$$

In Equation (3), *A* is a constant related to the transmission boundary, β is a modified Bessel function, and $\Gamma_{0}$ is the reflection coefficient at zero frequency:

(4) $$\Gamma_{0}=\frac{Z_{L}-Z_{0}}{Z_{L}+Z_{0}}\approx \frac{1}{2}\ln(\frac{Z_{L}}{Z_{0}})$$

The passband is defined as A; therefore, the maximum ripple in the passband is as follows:

(5) $$\Gamma_{m}=\frac{\Gamma_{0}}{\cosh A}$$

Regarding βL>A, Γ(θ) oscillates between $\pm \Gamma_{0}$/coshA.

**Figure 3:**
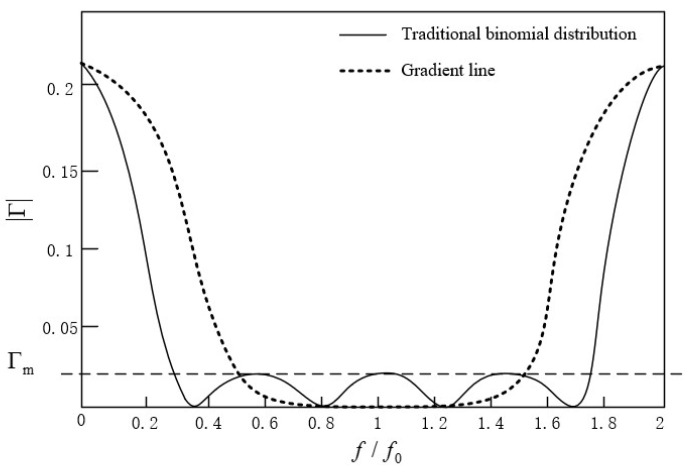
The relationship between the amplitude of the reflection coefficient and the frequency of the gradient line converter and traditional binomial distribution converter (fixed passband ripple of 0.02).

When the ripple in the passband was set to 0.02, the relationship curve between the amplitude of the reflection coefficient and the frequency of two matching converters with different structures was as shown in Figure 3.

In Figure 3, $f_{0}$ is the center frequency, f is the spectral width of the left and right frequency points corresponding to a 3 dB drop in insertion loss at the center frequency $f_{0}$, and $f/f_{0}$ is the relative bandwidth.

It can be seen that the gradient line had the same characteristics as the Chebyshev matching transformer. Although the passband was not as flat as the binomial multi-section matching converter, it had an equal ripple effect. Therefore, it was easier to control the reflection coefficient in the passband, and the working bandwidth was wider.

**Figure 4:**
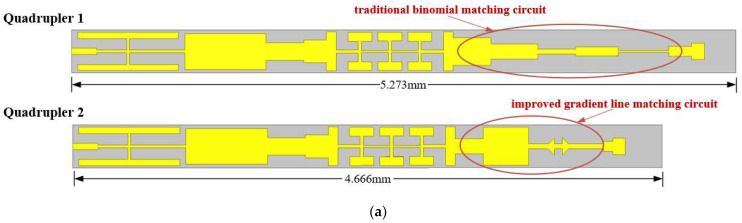

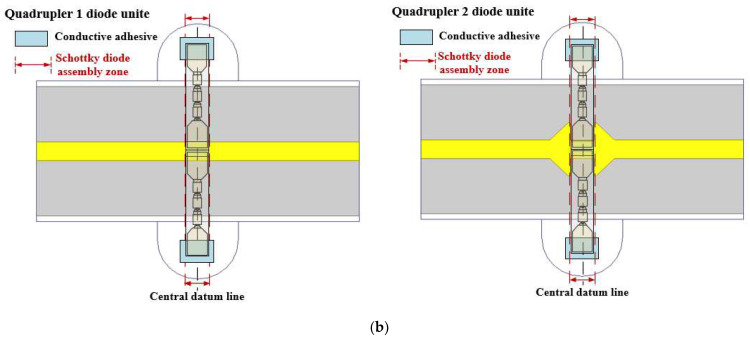
(**a**) Comparison of circuit structures; (**b**) comparison of diode units.

On the basis of the theoretical research of the tapered transmission line, the gradient structure was added to the design of the frequency quadrupler matching circuits. To verify the superiority of the gradient structure, a total of two frequency multipliers with different matching structures were designed. The traditional binomial matching structure and gradient line matching structure were correspondingly adopted in the two frequency quadruplers; the circuit comparison is shown in Figure 4a. It can be seen that the circuit using the gradient line matching structure was nearly 0.61 mm shorter than the traditional binomial distribution matching structure. It is worth mentioning that the position of this gradient line was at the connection between the diode and the transmission line, which can play a role in positioning the diode assembly. In summary, this can reduce the power loss by reducing the assembly error of the diode. A comparison of the diode units of two frequency quadruplers with different matching structures is shown in Figure 4b.

### 2.3. Overall Models and Simulation Results

**Figure 5:**
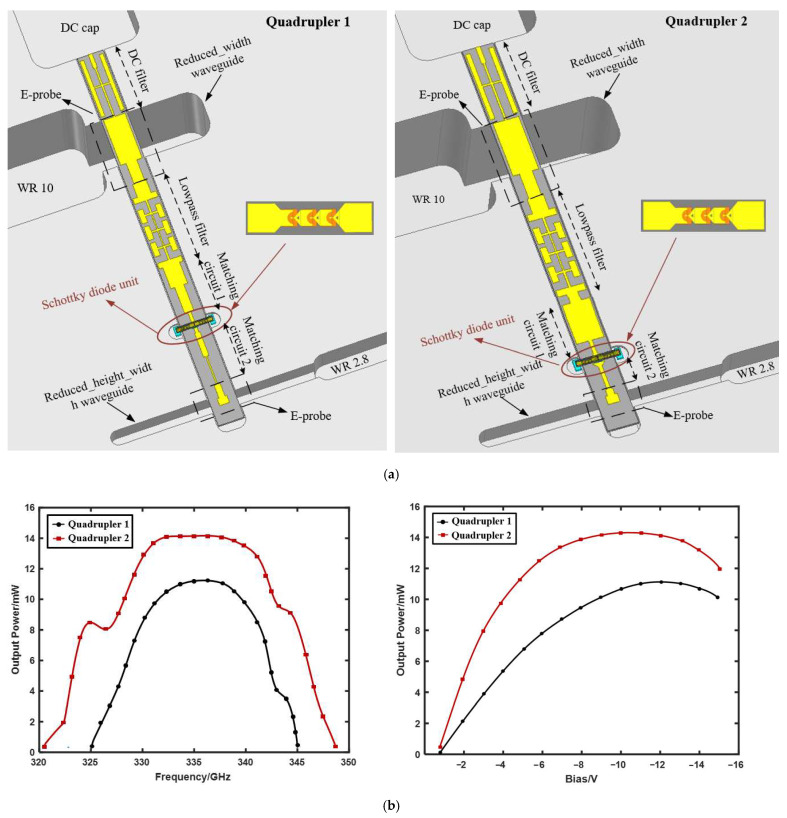
(**a**) Overall simulation model comparison of Quadrupler 1 and Quadrupler 2; (**b**) comparison of the simulation results of Quadrupler 1 and Quadrupler 2.

The overall models of the two frequency quadruplers included the waveguide–microstrip transition, a DC bias filter, a low-pass filter, a diode unit, and a matching circuit, which are shown in Figure 5a. The frequency multiplier of the traditional binomial matching structure is defined as Quadrupler 1, and the frequency multiplier of the gradient line matching structure is defined as Quadrupler 2. The DC bias filter was connected on the other side of the input waveguide in the same orientation as the diode circuit. The filter and the input waveguide–suspended microstrip transition formed a three-port network, which was similar to the design of a duplexer. This structure offers the possibility of forming the entire circuit on the same substrate only, and also achieves the separation of the diode circuit from the DC filter. Compared with the DC connection method using gold wire bonding, there was no movement of the connection position in this connection method, which greatly reduced the impact of assembly errors on the circuit suppression performance. The fundamental signal was input from the standard waveguide WR10 in TE10 mode, converted to TEM mode through the E-surface probe structure for transmission on the suspended microstrip line, and finally, output by the standard waveguide WR2.8. It is worth mentioning that in order to increase the coupling bandwidth, the input and output waveguides were reduced in width and height. The dimensions of the Quadrupler 1 input reduced_width waveguide and output reduced_height_width waveguide were 1.548 mm × 0.645 mm × 2.54 mm and 2.73 mm × 0.2 mm × 0.51 mm, respectively. The corresponding dimensions of Quadrupler 2 were 1.248 mm × 0.645 mm × 2.54 mm and 2.265 mm × 0.2 mm × 0.51 mm, respectively.

Figure 5b presents a comparison of the simulation results of the two quadruplers. It can be seen that the output power of the frequency multiplier with the gradient line matching structure was greater than that of the traditional binomial matching structure, and the bandwidth was widened by nearly 10 GHz. This is consistent with theoretical analysis [16,17]: at the same length, the gradual line structure can achieve the minimum reflection under a given length; in contrast, at a given reflection, the length of the transformation section required by the gradual line structure is the shortest. In other words, the latter can obtain a better and wider bandwidth than the former.

## 3. Fabrication and Measurement

The two designed quadruplers were machined and assembled, and a test system was built for testing.

### 3.1. Assembly and Fabrication

**Figure 6:**
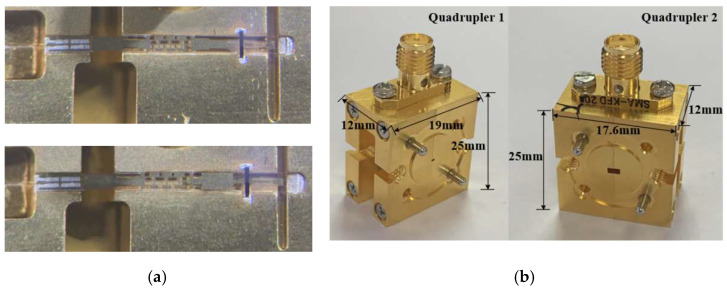
(**a**) Circuits in the quadrupler blocks; (**b**) photographs of the entire quadrupler modules.

Photographs of Quadrupler 1 and Quadrupler 2 are shown in Figure 6. The circuits of the quadruplers were integrated on a 50 µm thick quartz substrate and packaged in the blocks, which were split from the E-plane and fixed with screws. The blocks also integrated an SMA connector and standard flanges. The dimensions of quadruple multiplier modules 1 and 2 were 12 mm × 19 mm × 25 mm and 12 mm × 176 mm × 25 mm, respectively.

### 3.2. Test Results

**Figure 7:**
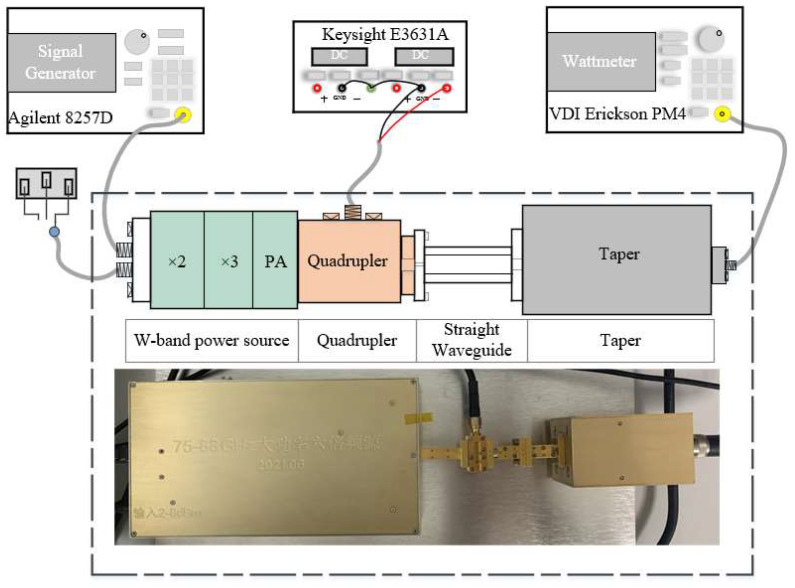
Test diagram of the 335 GHz quadrupler.

The block diagram of the measurement setup is illustrated in Figure 7. The Agilent analog signal generator E8257D (Santa Clara, CA, USA) and W-band power source were used to generate the signal in the 80–87 GHz band. The output power of the frequency quadruplers was measured using a PM4 power meter (Charlottesville, VA, USA). In addition, the SMA (Sub-Miniature-A) was connected to the main transmission circuit via gold wire bonding, and the DC source was connected to the SMA port to provide bias voltage for the frequency quadrupler.

**Figure 8:**
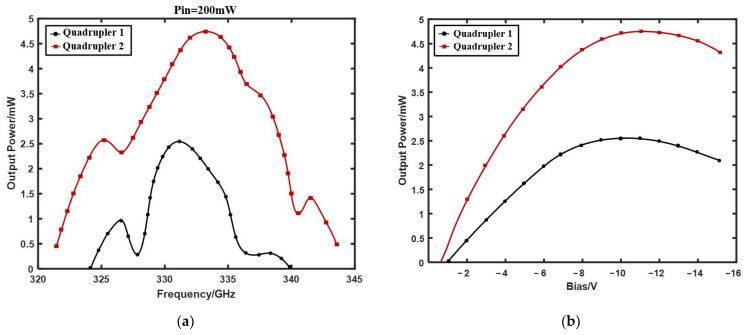

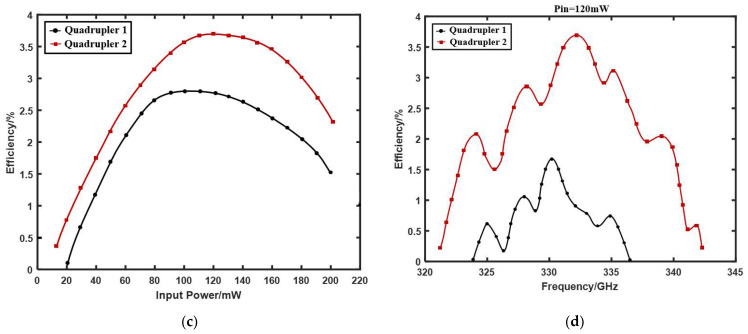
(**a**) Output power vs. frequency (fixed input power of 200 mW); (**b**) output power vs. bias (Quadrupler 2 and Quadrupler 2 at 333 GHz); (**c**) efficiency vs. input power (Quadrupler 2 and Quadrupler 2 at 333 GHz); (**d**) efficiency vs. frequency (fixed input power of 120 mW).

The test results are shown in Figure 8. Considering that the device could burn out due to excessive input power, the maximum input power of the quadrupler was fixed at 200 mW by adjusting the signal generator and the W-band power source. Due to the non-balanced circuit structure of the frequency multiplier, the efficiency is related to three variables: frequency, bias voltage, and input power. A comparison of the output power for the two 335 GHz quadruplers at 200 mW input power is shown in Figure 8a. The measured output power of the Quadrupler 2 was more than 0.5 mW at 321–344 GHz, and the maximum output power was approximately 4.75 mW at 333 GHz. Compared with Quadrupler 2, Quadrupler 1 had a narrower bandwidth with a maximum output power of 2.54 mw at 331 GHz. Figure 8b shows the relationship between the output power and bias voltage at 333 GHz for Quadrupler 1 and Quadrupler 2. The output power of the two quadruplers reached a maximum at bias voltages of −10.9 V and −10 V, respectively, and then began to decline. Figure 8c shows the efficiency versus input power at 333 GHz for Quadrupler 1 and Quadrupler 2. It can be seen that the efficiency increased with the increase in input power, until it reached the maximum at a certain input power and tended to be flat because the diode was close to saturation at this time. When the input power exceeded a certain value, the efficiency decreased with the increase in input power. The test results were basically consistent with the theoretical analysis. Based on the results in Figure 8c, the input power was adjusted to 120 mW, and the relationship between the efficiency and frequency of the two quadruplers is shown in Figure 8d. The results showed that Quadrupler 2 not only had a higher efficiency than Quadrupler 1, but also had a wider bandwidth.

Table 1 illustrates a comparison of some reported multipliers above 300 GHz. In addition, this study used discrete circuits to design the two quadruplers, which is easier to realize and relatively less expensive than integrated circuits. It can be seen that the measured results were lower than the simulated values, which may be due to the following reasons: (1) due to the higher frequency band, the impacts of machining error and assembly accuracy on the performance of the frequency multiplier cannot be ignored; (2) the presence of high-frequency physical effects in the diode affects the operating characteristics, resulting in the deterioration of performance.

## 4. Conclusions

A design method of an improved frequency multiplier matching circuit has been proposed in this paper. Based on this method, the traditional binomial matching structure frequency quadrupler and the improved gradient line matching structure frequency quadrupler were designed, fabricated, assembled, and measured. The improved design can effectively reduce the design size of the multiplier. The circuit using the gradient line matching structure was nearly 0.61 mm shorter than the traditional matching binomial distribution structure. The gradient line part can play a critical role in the assembly positioning of the diode, thus reducing the error introduced in manual assembly. The improved design improved the performance deterioration of the frequency multiplier caused by an excessively long substrate and assembly error, thereby improving the overall performance of the frequency multiplier. The measured typical output power of the improved frequency quadrupler was 2 mW at 324–339 GHz, and the peak measured output power was 4.75 mW at 333 GHz. The circuit length was reduced by nearly 0.61 mm compared with the conventional design frequency quadrupler, whereas the output power was nearly doubled, and the bandwidth was increased by 8 GHz. 

## Figures and Tables

**Table 1:** Performance comparison of the frequency multipliers working above 330 GHz.

References	Diode Style	Multiply Factor	Frequency	Input Power	Peak Output Power	FBW
[14] from CAEP (China Academy of Engineering Physics)	Discrete	4	332–345 GHz	200	4 mW at 339 GHz	335–344 GHz
[18] from VDI	Integrated	2	220–330 GHz	20–50	3 mW	-
[19] from JPL	Integrated	3	260–340 GHz	100	7.5 mW at 325 GHz	290–330 GHz
[20] from RAL	Discrete	2	329–338 GHz	25	1.2 mW at 332.8 GHz	329–337 GHz
[21] from UESTC (University of Electronic Science and Technology of China)	Discrete	3	320–342 GHz	10–42	0.149 mW at 326.5 GHz	320–335 GHz
This paper	Discrete	4	321–344 GHz	200	4.75 mW at 333 GHz	324–339 GHz

## Data Availability

Not applicable.
